# Chemokine Ligands and Receptors Regulate Macrophage Polarization in Atherosclerosis: A Comprehensive Database Mining Study

**DOI:** 10.1016/j.cjco.2024.11.018

**Published:** 2024-11-26

**Authors:** Wanqian Yu, Linghua Fu, Guangtao Lei, Fan Luo, Peng Yu, Wen Shen, Qinghua Wu, Pingping Yang

**Affiliations:** a Department of Cardiovascular Medicine, The Second Affiliated Hospital, Jiangxi Medical College, Nanchang University, Nanchang, Jiangxi, China; b Department of Gastroenterology, Jiangxi Provincial Hospital of Traditional Chinese Medicine, Nanchang, Jiangxi, China; c Department of Endocrinology and Metabolism, The Second Affiliated Hospital, Jiangxi Medical College, Nanchang University, Nanchang, China

## Abstract

**Background:**

Atherosclerosis is a systemic disease involving multiple blood vessels and a major cause of cardiovascular disease. Current treatment methods (eg, statins) for atherosclerosis can reduce the risk of cardiovascular diseases effectively, but they are insufficient to completely reverse existing atherosclerosis. Macrophages play a central role in development of atherosclerosis. Chemokines, the main mediators of macrophage chemotaxis, are important in immune and inflammatory responses. The effects of chemokines on mechanisms involved in atherosclerosis are unknown. This study preliminarily investigated these effects and mechanisms via bioinformatics methods.

**Methods:**

In this study, data on chemokine ligands and receptors were obtained by mining public databases (the National Center of Biotechnology Information-Gene Expression Omnibus [NCBI-GEO] database, ArrayExpress database, and single-cell RNA sequencing [scRNA-seq] database), and an extensive literature search was performed. The expression levels of chemokines in mouse tissues were analyzed via Metascape software for signalling pathway enrichment, scRNA-seq data for chemokine expression in atherosclerotic plaque progression and regression, and GEO2R data for chemokine expression during macrophage polarization. Ingenuity Pathway Analysis (IPA) software was used to analyze regulatory factors such as transcription factors and microRNAs that are significantly differentially expressed upstream of chemokines in macrophage polarization. Finally, a model of the chemokine regulation of atherosclerosis was established on the basis of these results.

**Results:**

There are 5 main findings: (1) In atherosclerosis, chemokines are regulated by transcription factors and microRNAs. (2) The transcription factor STAT1 promotes the polarization of dormant (M0) macrophages into classically activated (M1) macrophages and alternative activated (M2) macrophages by regulating chemokines. The transcription factors STAT1, IRF7 and IRF1 regulate the polarization of M0 macrophages into M2a and M2b macrophages via different chemokines. For example, some transcription factors promote M1 polarization of M0 macrophages through CCL4, but M2 macrophage polarization is regulated via CCL19, CCL5 and CCR7. (3) Transcription factors can promote and inhibit, whereas miRNAs can only inhibit atherosclerosis. (4) CCL4 existed in all 5 different chemokine-regulated macrophage models, whereas CXCL3 only existed in the M2b macrophage transcriptional regulation model, indicating that CXCL3 may promote the M2b type macrophages polarization of M0 macrophages. (5) CCL5 and CCR7 can promote the M2a macrophages and M2b macrophages polarization of M0 macrophages.

**Conclusions:**

Atherosclerosis can be treated by regulating chemokines and regulating the polarization of macrophages. The chemokines CCL4, CCL5, CCL8, CCL19, CXCL3, CXCL10, CXCL13, and CCR7 may play key roles in the progression and regression of atherosclerosis.

## Résumé

**Contexte:**

L’athérosclérose, une affection généralisée qui touche de nombreux vaisseaux sanguins, est une des principales causes de maladies cardiovasculaires. Les traitements actuels (p. ex. les statines) permettent de réduire efficacement le risque de maladie cardiovasculaire, mais ils ne permettent pas à eux seuls de renverser complètement l’athérosclérose. Les macrophages jouent un rôle central dans l’apparition de l’athérosclérose. Les chimiokines, principaux médiateurs du chimiotactisme des macrophages, jouent un rôle important dans les réponses immunitaires et inflammatoires. Les effets des chimiokines sur les mécanismes qui interviennent dans l’athérosclérose ne sont pas connus. La présente étude préliminaire a examiné ces effets et mécanismes par des méthodes bio-informatiques.

**Méthodologie:**

Dans cette étude, les données relatives aux ligands et aux récepteurs des chimiokines ont été obtenues par une recherche effectuée dans des bases de données publiques (National Center of Biotechnology Information-Gene Expression Omnibus [NCBI-GEO], ArrayExpress et une base de données scRNA-seq de transcriptomes de cellules uniques) de même que dans le cadre d’une revue exhaustive de la littérature médicale. Les chercheurs ont analysé le degré d’expression des chimiokines dans le tissu murin à l’aide ArrayExpress de Metascape pour l’enrichissement des voies de signalisation, et procédé à l’analyse du scRNA-seq pour mesurer l’expression des chimiokines dans la progression et la régression de la plaque athéromateuse; les données GEO2R ont permis de mesurer l’expression des chimiokines durant la polarisation macrophagique. Le logiciel Ingenuity Pathway Analysis (IPA) a servi à analyser les facteurs de régulation, comme les facteurs de transcription et les microARN, dont l’expression varie de façon marquée en amont de la polarisation des macrophages par les chimiokines. Finalement, un modèle de régulation des chimiokines dans l’athérosclérose a été mis au point à partir de ces résultats.

**Résultats:**

Cinq constatations principales ont été dégagées : 1) Dans l’athérosclérose, la régulation des chimiokines repose sur les facteurs de transcription et les microARN. 2) Le facteur de transcription STAT1 favorise la polarisation des macrophages en dormance (M0) en phénotype M1 (macrophages activés par la voie classique) et en phénotype M2 (macrophages activés par d’autres voies) par les chimiokines régulatrices. Les facteurs de transcription STAT1, IRF7 et IRF1 régulent la polarisation des macrophages M0 en phénotypes M2a et M2b par différentes chimiokines. Par exemple, certains facteurs de transcription favorisent la polarisation des macrophages M0 en phénotype M1 par le CCL4, mais la polarisation des macrophages M2 est régulée par CCL19, CCL5 et CCR7. 3) Les facteurs de transcription peuvent favoriser ou inhiber l’athérosclérose, alors que les microARN peuvent seulement inhiber la progression de l’athérosclérose. 4) Le CCL4 était présent dans les cinq différents modèles de macrophages régulés par les chimiokines, alors que le CXCL3 était présent uniquement dans le modèle de régulation transcriptionnelle des macrophages M2b, ce qui indique que CXCL3 pourrait favoriser la polarisation des macrophages M0 en phénotype M2b. 5) Les CCL5 et CCR7 peuvent favoriser la polarisation des macrophages M0 en phénotypes M2a et M2b.

**Conclusions:**

La régulation des chimiokines et la régulation de la polarisation des macrophages peuvent permettre de traiter l’athérosclérose. Les chimiokines CCL4, CCL5, CCL8, CCL19, CXCL3, CXCL10, CXCL13 et CCR7 pourraient jouer un rôle clé dans la progression et dans la régression de l’athérosclérose.

Atherosclerosis (AS) is a systemic disease involving multiple arteries and is a major cause of cardiovascular disease.^1^^,^^2^ It involves a chronic inflammatory process characterized primarily by the localized deposition of lipoproteins in arterial walls. The cholesterol and oxidized phospholipids within these lipoproteins trigger endothelial cell activation.^3^^,^^4^ Activated endothelial cells recruit monocytes into the intima and subintima of the arteries,^5^^,^^6^ where monocytes further differentiate into proinflammatory macrophages.^7^ These macrophages engulf cholesterol-rich lipids and transform into foam cells, which contribute to the development of atherosclerotic plaques: an important hallmark of both early and advanced AS lesions.^8^, ^9^, ^10^ However, at present, AS is still not reversible by pharmacologic treatment. Therefore, a better understanding of the pathogenesis of plaque formation is needed for the future development of new targets or therapeutic strategies to combat this disease.

Macrophages are a highly heterogeneous and plastic cell population that differentiates from monocytes as they migrate into tissues.^11^ These cells can polarize into 2 main phenotypes: proinflammatory M1-type macrophages and anti-inflammatory M2-type macrophages. M2 macrophages are further divided into M2a, M2b, and M2c subtypes, each of which plays distinct roles in tissue repair.^12^ M2a and M2b macrophages are involved in immunomodulation and the promotion of the M2-type immune response, whereas M2c macrophages contribute to immune suppression and tissue remodelling.^13^ During atherogenesis, macrophages exhibit various polarization states in response to the plaque microenvironment,^14^ which leads to distinct gene and protein expression profiles.^15^ Studies have shown that M1-type macrophages promote the formation of unstable plaques, whereas M2-type macrophages reduce plaque size and increase stability, potentially preventing progression of AS.^16^, ^17^, ^18^ Therefore, modulating macrophage polarization represents a promising strategy for treatment of AS. Numerous factors, such as chemokines, microRNAs (miRNAs), and transcription factors (TFs), influence macrophage polarization; however, the precise mechanisms by which they regulate this process remain unclear and require further study.

Chemokines, a class of small-molecule proteins, are key mediators of macrophage polarization and play critical roles in immune and inflammatory responses.^19^^,^^20^ Chemokines are classified into 4 isoforms on the basis of the number and position of conserved cysteine residues at their N termini: CC, CXC, CX3C, and XC.^21^^,^^22^ Their biological activity is mediated by binding to specific 7-transmembrane G protein-coupled receptors (GPCRs) expressed on target cells.^23^ Initially, chemokines were found to direct leukocytes to sites of inflammation by binding to chemokine receptors. Recent studies, however, have revealed that chemokines are not only involved in cell recruitment but also highly expressed in cells crucial to development of AS, including endothelial cells, smooth muscle cells, and macrophages. For example, Xuan et al.^24^ demonstrated that chemokines such as CCL19, CCL21, CCL24, CCL25, CXCL8, CXCL10, and XCL2 specifically induce M1 macrophage polarization, whereas CCL7 induces both M1 and M2 macrophages. Chemokines play pivotal roles throughout the progression of atherogenesis.^25^ Combadière et al.^26^ reported that reducing the levels of CCL2, along with the chemokine receptors CCR5 and CX3CR1, significantly decreased the number of macrophages within atherosclerotic plaques. In addition, studies by Boring^27^ and Gu^28^ suggested that CCL2 and CCR2 regulate AS by influencing macrophage migration. These findings indicate that chemokines may control several processes involving monocyte-derived macrophages within atherosclerotic lesions.

MiRNAs are noncoding RNAs approximately 22 nucleotides long that act as post-transcriptional regulators of gene expression and can modulate progression of AS by influencing chemokine expression.^29^ For example, miRNA-155 promotes AS by directly inhibiting bcl6 (a TF that attenuates proinflammatory NF-κB signalling), thereby increasing CCL2 expression in M1 macrophages.^30^ In contrast, miRNA-467b suppresses progression of AS by downregulating macrophage lipoprotein lipase (LPL) and CCL2 expression.^31^ TFs, which recognize specific DNA sequences, control chromatin remodelling and gene transcription, playing essential roles in development of AS.^32^, ^33^, ^34^, ^35^ For example, NF-κB has been shown to promote AS by increasing CXCL8 release.^36^ Although the direct regulation of macrophage polarization via chemokines by miRNAs and TFs has yet to be fully demonstrated, the evidence suggests that both can influence progression of AS by modulating chemokine activity. We hypothesize that miRNAs or TFs may regulate AS through chemokine-mediated signalling pathways.

Our current study aims to elucidate the mechanisms by which chemokines and their receptors induce macrophage polarization during AS via bioinformatics analysis of large public datasets and a thorough review of the literature. We seek to establish a regulatory model that outlines the role of chemokines in the development of AS.

## Materials and Methods

### Chemokine genes and database mining strategies

**Figure 1:**
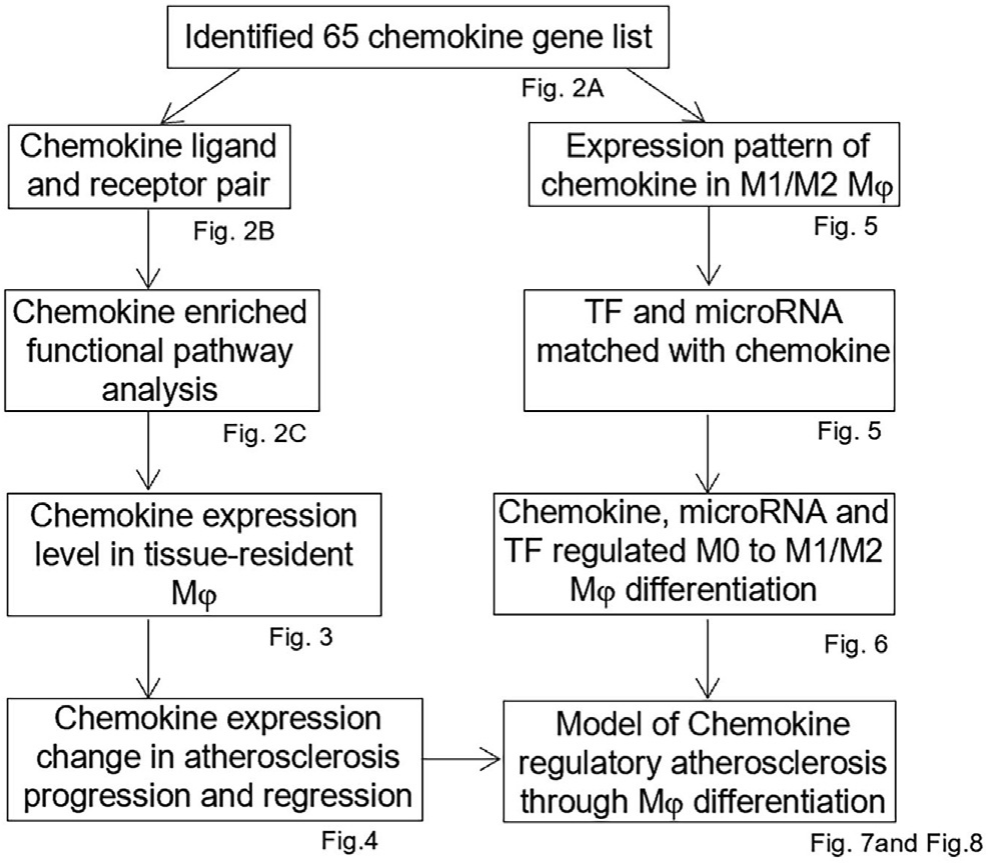
The overall strategy of the identification of chemokine signalling in macrophage subset polarization and atherosclerosis processes.

**Figure 2:**
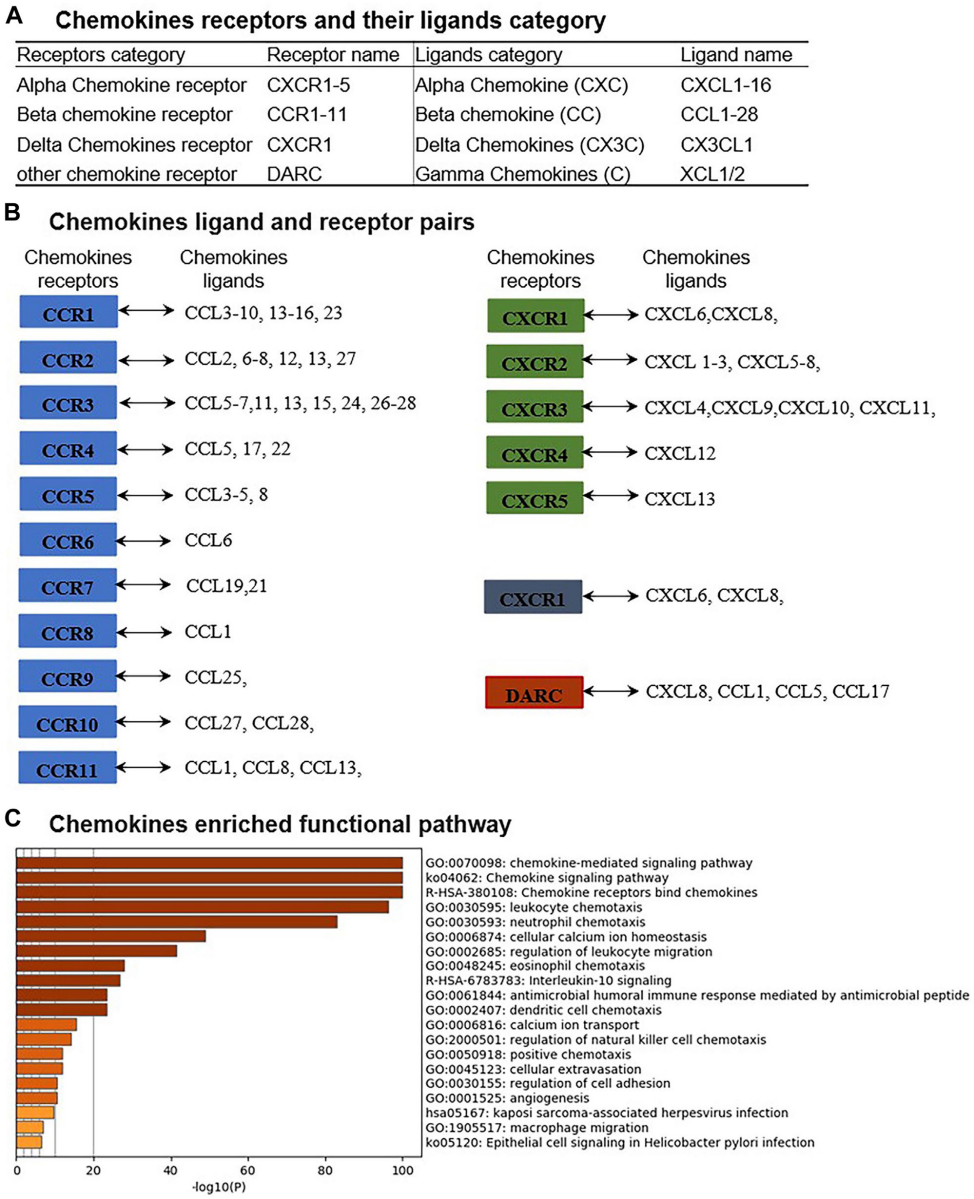
Chemokines gene identified and paired and functional pathway. (**A**) Chemokines receptors and their ligands category. Sixty-five chemokine genes were identified by a literature search. (**B**) Chemokines ligand and receptor pairs.(**C**) Chemokines enriched functional pathway. Chemokine ligand paired with chemokine receptor and were characterized functional pathway by using Metascape software.

The overall strategy is summarized in Figure 1. We selected 65 chemokine genes via the National Institutes of Health (NIH) and National Center of Biotechnology Information (NCBI) Unigene databases (https://www.ncbi.nlm.nih.gov/gene/), the results of which are listed in Figure 2A. We used Metascape software to pair chemokine ligands with chemokine receptors (Fig. 2B) and investigate their functional pathways. We established tissue-specific expression patterns of chemokines in macrophages and explored changes in chemokine expression during progression and regression of AS. In addition, we explored the expression patterns of chemokines in M1/M2 macrophages and used Ingenuity Pathway Analysis (IPA, http://www.ingenuity.com/) to match significantly differentially expressed (SDE) chemokines with SDE TFs and miRNAs. Finally, we established a chemokine regulatory AS model via macrophage polarization and verified its reliability via a literature search.

### Atherosclerosis and macrophage polarization

Atherosclerosis is a progressive disease characterized by chronic inflammation and lipid accumulation in the arterial wall. The development of atherosclerotic plaques is closely associated with macrophage infiltration and activation. Macrophages derived from recruited monocytes exhibit high plasticity and can polarize into various functional states, primarily the proinflammatory M1 and anti-inflammatory M2 subtypes. Notably, M2 macrophages can be further categorized into M2a, M2b, and M2c subtypes based on their distinct functions in tissue repair and immune modulation.^12^ Previous studies have demonstrated that M1 macrophages promote plaque instability and lesion progression, whereas M2 macrophages, particularly M2a and M2b macrophages, contribute to plaque stabilization and regression by promoting anti-inflammatory responses and resolving inflammation.^16^, ^17^, ^18^

### Tissue-specific macrophages expression pattern of chemokines

In this study, a data ID: experiment E-GEOD-63340 (Table 1**)**^37^ was screened from the matrix permutation database (ArrayExpress, https://www.ebi.ac.uk/arrayexpress/) from the European Association of Bioinformatics by searching the following keywords: macrophage, mouse, and RNA sequencing. Then we analyzed the chemokine expression information in tissue characteristic macrophages by ArrayXpress. Chemokine mRNA levels are described as gene transcript units per million transcripts (TPM). The functional role of chemokines in macrophage polarization was examined in detail by exploring their expression patterns across tissue-specific macrophages from mice.

**Table 1:** Macrophage and chemokine data sources

Author	Year	Cell species	PMID
Lavin	2014	Mouse	25480296
Lin	2019	Mouse	30830865
Fujiwara	2016	Human	27990286

### Chemokine expression changes in progression and regression of AS

We first obtained the data (GSE123587) (Table 1**)**^38^ in the single-cell RNA sequencing (single-cell RNA sequencing, scRNA-seq) database by searching "scRNA-seq" and "macrophages," and then we used scRNA-seq to analyze the role of chemokine expression from macrophage precursors in plaque macrophages in the progression and regression of AS.

### Expression pattern of chemokines in M1/M2 macrophages

In this study, the data (GSE85346) (Table 1**)**^12^ were obtained by searching the keyword "macrophage polarization" in the NIH-NCBI-Geo database, and then the expression of chemokine during macrophage polarization was analyzed by GEO2R. Numbers with a red-coloured background indicate fold change > 2 (log_2_ FC > 1). Numbers with a green-coloured background indicate fold change < 0.5 (log_2_ FC < –1).

### TFs, miRNAs, and chemokines matching

Emerging evidence suggests that chemokines not only are key mediators of immune cell recruitment but also influence macrophage polarization by interacting with upstream regulatory factors such as TFs and miRNAs. Using IPA, we identified significantly differentially expressed TFs and miRNAs that modulate chemokine expression, affecting macrophage phenotypes and the course of AS. The transcriptional regulatory relationship between SDE TF and SDE chemokines was considered significant when the *P* value < 0.01 and the |z score| > 2. SDE chemokines were matched with miRNA by IPA upstream analysis. The transcriptional regulatory relationship between miRNA and SDE chemokines was considered significant when the *P* value < 0.01 and |z score| > 2.

### Chemokines, miRNAs, and TFs regulate M0 to M1/M2 macrophage polarization

We combined the expression pattern of chemokines in macrophage polarization with the upstream molecules (TFs and miRNAs) corresponding to chemokines obtained by IPA to obtain the signalling pathways of chemokines, TFs, and miRNAs regulating macrophage polarization.

### Establishment of a model of chemokine regulation of AS through macrophage polarization

A central aim of our study was to construct predictive models illustrating how chemokines regulate macrophage polarization in the context of AS. Through the integration of gene expression data and literature-based evidence, we constructed a model that describes the regulatory pathways of chemokines in macrophage activation. This model highlights how certain chemokines contribute to the progression of AS by promoting M1 polarization, whereas others facilitate regression by favouring M2 polarization. By matching the previously obtained chemokines, TFs, and miRNAs that regulate the macrophage polarization signalling pathway with the corresponding chemokines involved in the progression and regression of AS, we finally established the signalling pathway of the chemokine regulation of AS through macrophage polarization.

## Results

### Enrichment analysis of chemokine genes

To explore the interaction between chemokine ligands and their receptors, we first selected 65 chemokine genes through a literature search and matched their receptors and ligands using Metascape software. Then, we performed gene ontology (GO) and KEGG (Kyoto Encyclopedia of Genes and Genomes) pathway enrichment analyses on these genes using the Metascape database. To study the functional mechanism of chemokines in the development of AS, GO analysis was performed. The chemokine gene enrichment analysis reveals several key functional pathways associated with chemokine signalling. As shown in Figure 2A, chemokine receptors and their corresponding ligands are categorized into different classes: alpha chemokine receptors (CXCR1-5), beta chemokine receptors (CCR1-11), delta chemokine receptors (CXCR1), and other chemokine receptors such as DARC. The ligands of these proteins include various CXC, CC, CX3C, and XC chemokines, demonstrating the diversity of chemokine-receptor interactions that can regulate immune responses.

As shown in Figure 2B, the receptor-ligand pairing highlights how specific chemokine receptors bind to multiple ligands, with each receptor interacting with different sets of chemokines. For example, CCR1 interacts with CCL3, CCL5, and CCL23, whereas CCR5 binds to CCL3-5, emphasizing the complexity and redundancy of the chemokine signalling system. Other notable receptor-ligand interactions include those involving CXCR2, which binds to CXCL1-3 and CXCL5-8, playing a significant role in neutrophil chemotaxis and immune cell recruitment. Figure 2C displays the enriched functional pathways associated with chemokines. The results revealed that the target genes were associated mainly with the terms “chemokine-mediated signalling pathway,” “leukocyte chemotaxis,” and “neutrophil chemotaxis.” The target genes were enriched primarily in the "chemokine signalling pathway" and "chemokine receptors bind chemokines," according to the KEGG pathway analysis, further suggesting that chemokines are involved in a broad range of biological processes, from immune cell migration to tissue repair.

### Differential distribution of chemokines in mouse tissues

**Figure 3:**
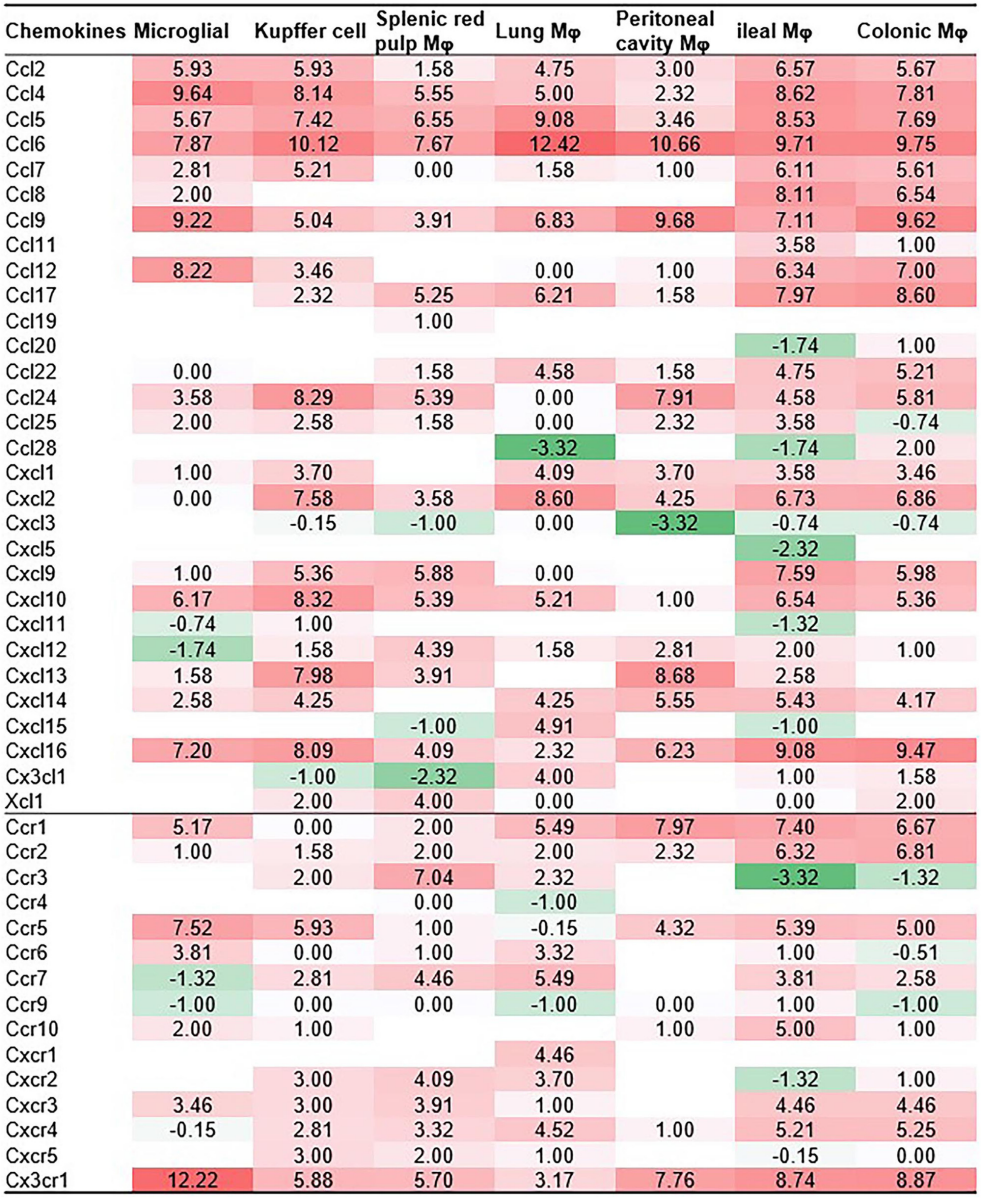
Chemokines expression level in tissue-specific macrophages (log_2_TPM) RNA-Seq datasets were collected from ArrayXpress of European Bioinformatics Institute, which stores data from high-throughput functional genomics experiments (https://www.ebi.ac.uk/arrayexpress). These data include information on the expression of T-cell costimulation receptors and coinhibition receptors through experiments submitted directly to ArrayXpress (PMID: 25480296). The **red colour** indicated log_2_TPM > 0, **green colour** background indicated log_2_TPM < 0.

Figure 3 shows the heatmap illustrating the differential expression of chemokines and chemokine receptors across various mouse macrophage (M0) populations, including microglia, Kupffer cells, splenic red pulp macrophages, lung macrophages, peritoneal cavity macrophages, ileal macrophages, and colonic macrophages. Chemokines such as CCL2, CCL4, and CCL5 are broadly expressed across multiple macrophage populations, with high levels observed in microglia, Kupffer cells, and colonic macrophages. For example, CCL4 expression is elevated in Kupffer cells (9.64), peritoneal macrophages (8.53), and colonic macrophages (7.69), indicating its role in these tissues. Similarly, CCL6 and Ccl7 are highly expressed in lung and peritoneal macrophages, suggesting a tissue-specific role in pulmonary and abdominal immune responses. In contrast, certain chemokines, such as CCL11 (eotaxin) and CCL17, have tissue-specific distributions and are highly expressed in microglia and Kupffer cells but less highly expressed in other macrophage populations. CCL25 is elevated in ileal macrophages (7.91), reflecting its known role in gut immunity. Among chemokine receptors, CCR5 and CX3CR1 are notably expressed across several tissues. CCR5 is highly expressed in microglia (7.52) and Kupffer cells (8.09), indicating its involvement in the central nervous system and liver macrophage function. CX3CR1, an important receptor for fractalkine signalling, is highly expressed in microglia (12.22) and moderately expressed in other tissues, reinforcing its role in neuroimmune interactions. Overall, the data demonstrate that chemokine and receptor expression varies widely between tissue-specific macrophage populations, suggesting functional specialization of chemokine signalling in different microenvironments such as the brain, liver, gut, and lungs.

### Chemokines are differentially expressed in the progression and regression of atherosclerotic plaques

**Figure 4:**
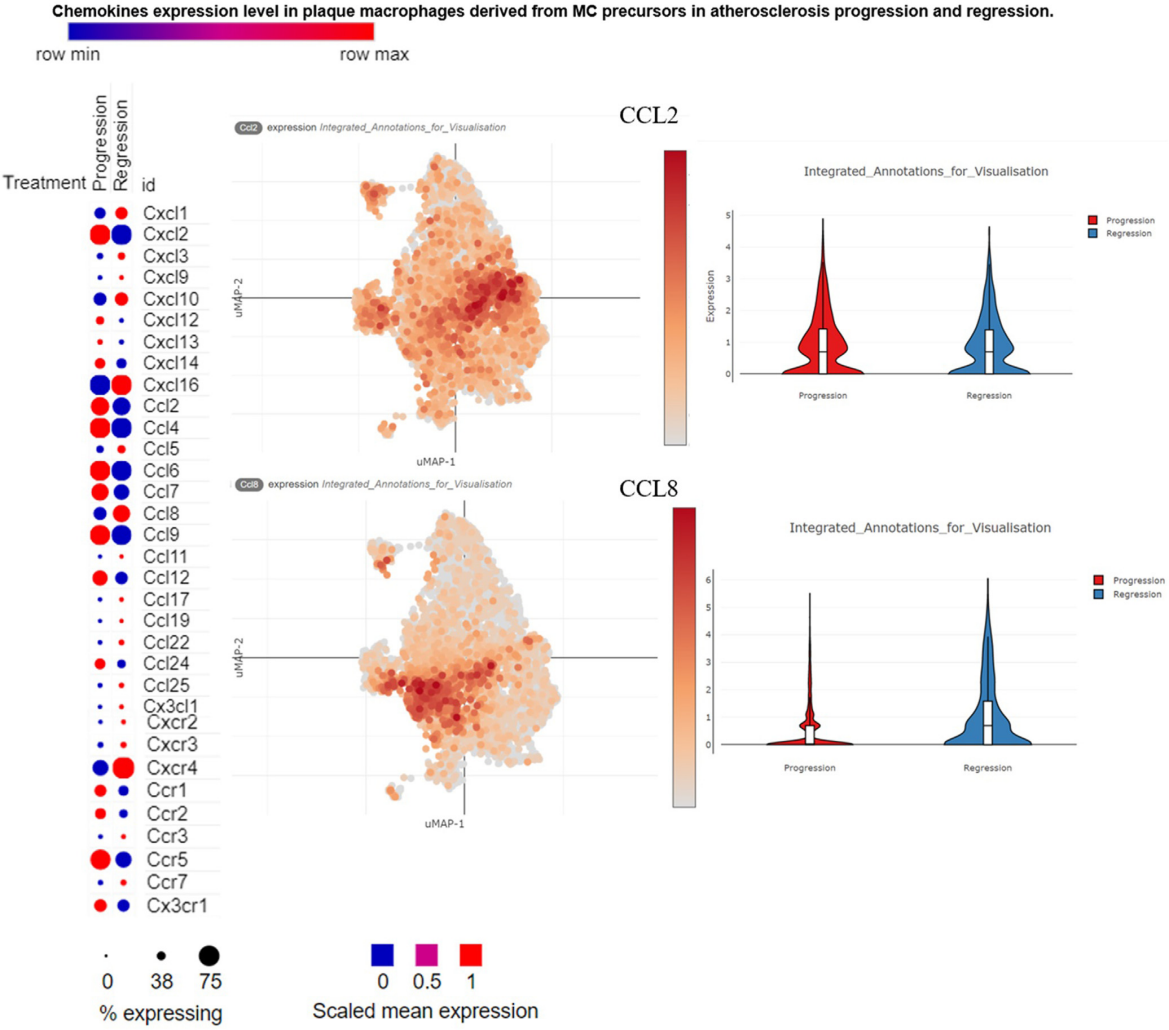
Chemokine expression change in atherosclerosis progression and regression.

We investigated the changes in the expression of 33 chemokines during the progression and regression of AS via scRNA-seq analysis (Fig. 4). The expression levels of various chemokines differ significantly between the progression and regression phases of AS. We defined the progression and regeneration of atherosclerotic plaques as follows: To analyze the phenotypic characteristics of cells derived from CX3CR1+ precursors during the progression and regression of AS, we employed a modified version of our recently reported model, avoiding the generation of bone marrow chimeras in Ldlr^–/–^ mice. Plaque progression is initiated by administering an adeno-associated viral vector that expresses a gain-of-function mutant of protein convertase subtilisin/kexin type 9 into Cx3cr1^CreERT2-IRES-YFP/+^Rosa26^fl-tdTomato/+^ mice. This approach leads to low-density lipoprotein (LDL) receptor deficiency and subsequent hypercholesterolemia. Once plaques are established, regression is induced by lowering plasma lipid levels through the administration of an antisense oligonucleotide targeting apolipoprotein B, which effectively reduces production of LDL.^38^

Ly6C^high^ monocytes play crucial roles in initiating and sustaining inflammatory responses. Upon exposure to inflammatory stimuli, these cells undergo metabolic reprogramming via oxidative phosphorylation to facilitate their migration and subsequent differentiation into macrophages. Once differentiated, Ly6C^hi^ monocytes exhibit distinct behaviours that depend on the tissue environment and the specific inflammatory signals present. In addition, our previous research revealed that chemokines and transcription factors could regulate the differentiation of Ly6C^high^ and Ly6C^low^ monocytes, thereby playing an anti-inflammatory and proinflammatory role.^39^

We used the size of the circular volume to represent the level of chemokine expression. In both processes, red represents an advantage, and blue represents a weakness. In general, chemokine expression tends to be greater during the progression phase than during the regression phase, indicating an active role in attracting immune cells to plaques during disease development. Chemokines such as CXCL2, CCL2, CCL4, and CCR5 are more highly expressed during progression of AS, as shown by the larger red circles on the heatmap and stronger expression signals in the corresponding violin plots. These findings suggest that these chemokines contribute to formation of plaque and inflammation. During regression, the expression of the same chemokines is markedly lower, suggesting a reduced inflammatory response, as shown by the larger red circles on the heatmap and stronger expression signals in the corresponding violin plots. These findings suggest that these chemokines contribute to plaque formation and inflammation. Chemokines such as CCL2, CCL4, and CCR5 are upregulated during the progression of AS, attracting Ly6C^hi^ monocytes to differentiate into M1 macrophages and promoting formation of plaque. During regression, chemokines such as CXCL16 and CCR7 become more prominent, signalling a shift toward resolution of inflammation and tissue repair. These results suggest that the chemokines CXCL2, CCL2, CCL4, CCL6, CCL7, CCL9, CCL12 and CCL24 tend to play a role in progression. Similarly, the chemokines CXCL16, CCL8, CCR7, and CXCR4 tended to play a role in regression.

### Expression of chemokines in differentiated macrophages

**Figure 5:**
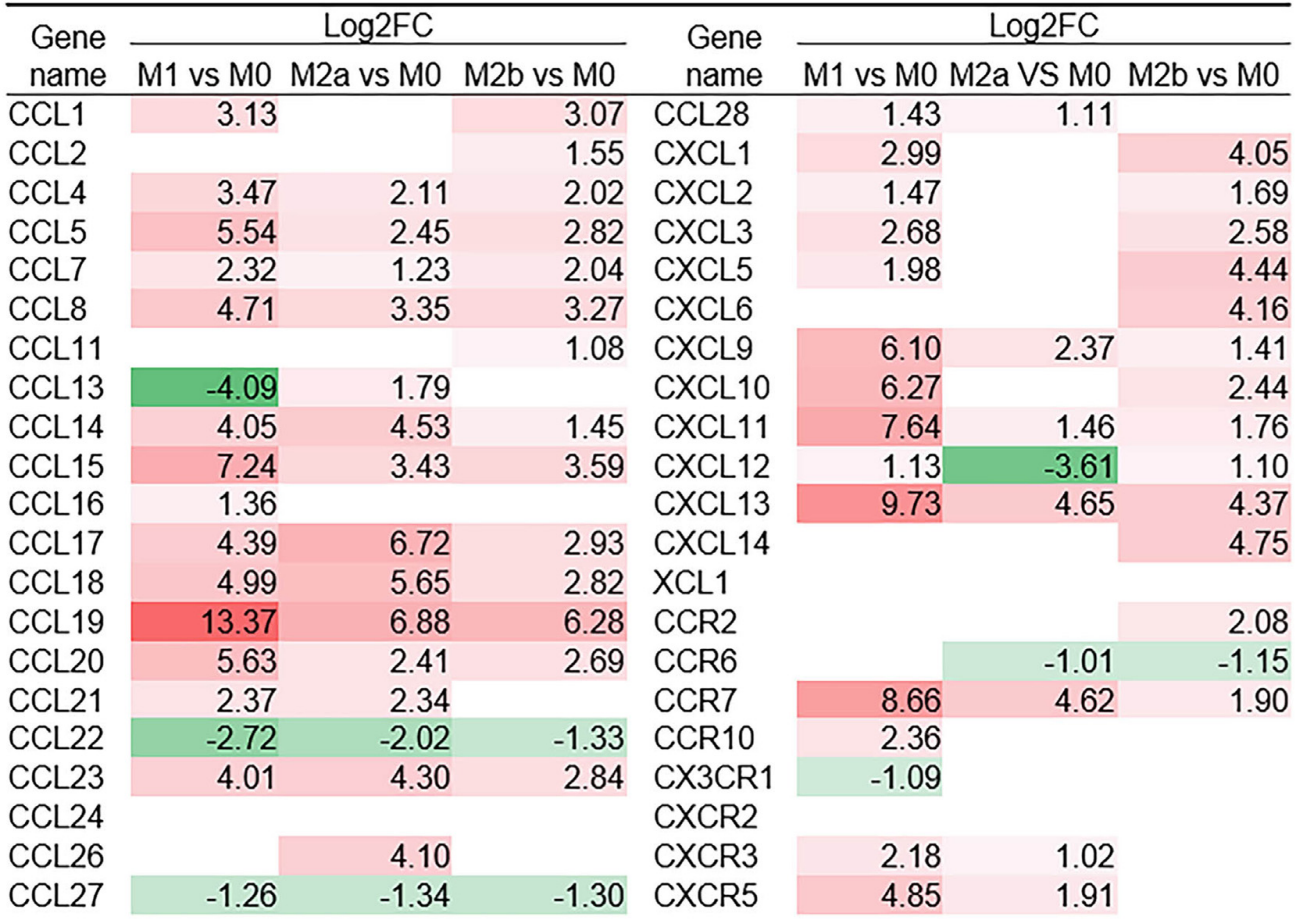
Chemokines expression level in macrophage polarization. Microarray datasets (GSE85346) collected from the NIH-NCBI-GEO Data Sets database and were analyzed in this study (PMID: 27990286). Numbers with **red-coloured** background indicate fold change > 2 (log_2_FC > 1). Numbers with **green-coloured** background indicate fold change < 0.5 (log_2_FC < –1). NIH-NCBI-GEO, National Institutes of Health-National Center of Biotechnology Information-Gene Expression Omnibus.

By using GEO2R analysis, we obtained the expression levels of 42 chemokines in M1- and M2-differentiated macrophages from mice and humans, as shown in Figure 5. The results are expressed as log 2-fold changes (log_2_FCs). The results revealed significant differential expression of various chemokines from the CCL family across macrophage phenotypes. Specifically, CCL1, CCL4, CCL5, CCL8, CCL13, CCL15, CCL17, CCL18, and CCL19 were notably upregulated in M1 macrophages, with CCL19 showing the greatest increase (log_2_FC = 13.37). Conversely, CCL11 was significantly downregulated in the M1 phenotype (log_2_FC = –4.09). In M2a macrophages, CCL17 and CCL26 were substantially upregulated, whereas CCL22 was notably downregulated. Among the CXCL chemokines, CXCL9, CXCL10, CXCL11, and CXCL13 were significantly upregulated in M1 macrophages, with the level of CXCL13 markedly increased (log_2_FC = 9.73). In contrast, CXCL12 was significantly downregulated in M2a macrophages (log_2_FC = –3.61). In addition, analysis of chemokine receptors revealed that CCR7 was markedly upregulated in M1 macrophages (log_2_FC = 8.66), whereas CCR2 expression was significantly lower in the M2a phenotype (log_2_FC = –1.01).

These findings underscore the distinct expression patterns of chemokines and receptors across macrophage phenotypes, particularly the pronounced upregulation of certain chemokines in M1 macrophages. These findings suggest that these chemokines may play a role in the functional specialization of macrophage subsets. For example, the expression of CCR7 and CCR2 is closely associated with M1 and M2 macrophages, respectively.

CCR7 (C-C chemokine receptor 7) is predominantly linked to M1 macrophages, which are characterized as "classically activated" or "proinflammatory." These macrophages participate in antimicrobial and antitumour immune responses. Elevated CCR7 expression facilitates the migration of M1 macrophages to lymph nodes, thereby increasing antigen presentation and T-cell activation, which are essential for initiating and maintaining an effective immune response. These findings underscore the role of CCR7 in promoting immune activation and inflammation.

In contrast, CCR2 (C-C chemokine receptor 2) is associated with M2 macrophages, which are involved in anti-inflammatory responses and tissue repair. The differential expression of CCR7 and CCR2 between M1 and M2 macrophages indicates their distinct roles in the functional specialization of macrophage subsets, suggesting a regulatory mechanism that balances proinflammatory and anti-inflammatory responses.

### Identification of miRNAs and SDE TFs and establishment of a model for chemokine regulation of AS through macrophage polarization

**Figure 6:**
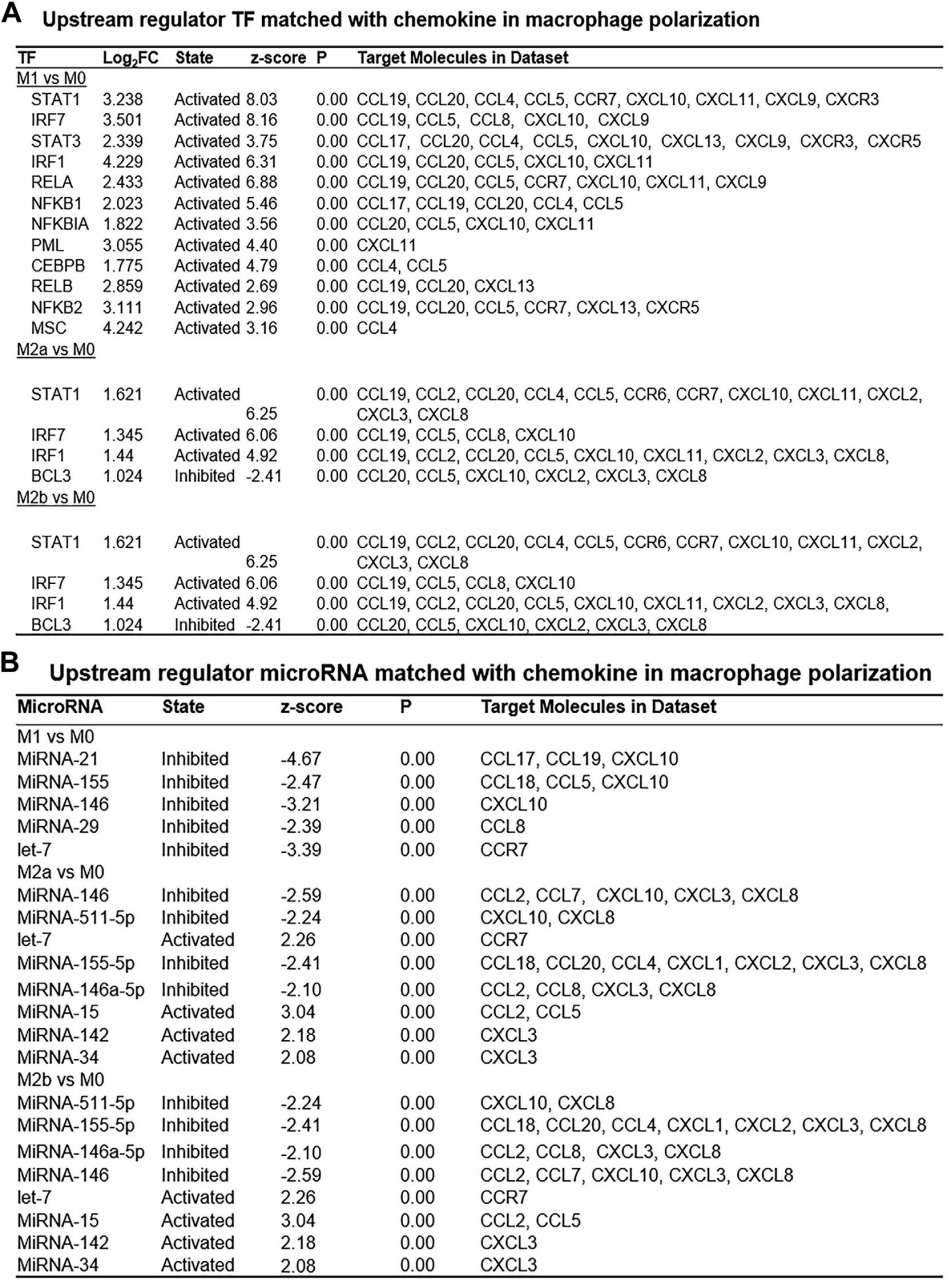
Chemokine, transcription factors (TFs) and microRNAs (miRNAs) in macrophage polarization. (**A**) Upstream regulator TF matched with chemokine in macrophage polarization. Significant differential expression (SDE) chemokines were matched with SDE TF by IPA upstream analysis. Transcriptional regulatory relationship between SDE TF and SDE chemokines was justified by *P* value < 0.01 and |z-score | > 2. (**B**) Upstream regulator microRNA matched with chemokine in macrophage polarization. SDE chemokines were matched with microRNA by IPA upstream analysis. Transcriptional regulatory relationship between microRNA and SDE chemokines was justified by *P* value < 0.01 and |z score |> 2.

**Figure 7:**
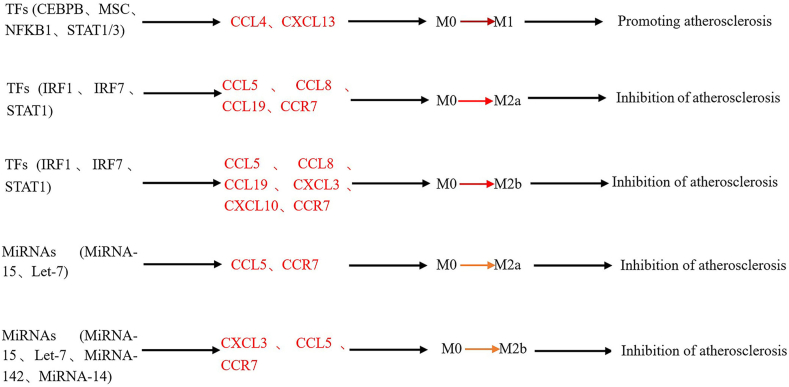
Chemokine, microRNA, and TF regulated M0 to M1/M2 macrophage polarization. TF, transcription factor.

To identify the potential transcriptional regulatory axis involved in macrophage polarization, miRNAs and SDE TFs were matched with corresponding downstream immunologic SDE chemokines via IPA upstream analysis. We found that 10 miRNAs and 13 SDE TFs were positively correlated with various downstream SDE chemokines (Fig. 6, A and B). These pathways represent potential transcriptional regulatory mechanisms that could determine differential immunologic features and subset polarization. Two representative miRNAs and SDE TFs were chosen to further elucidate their relevant transcriptional regulatory pathways. STAT1, a major member of the STAT family, is involved in the regulation of inflammation, mainly through the formation of homodimers and their translocation to the nucleus.^40^, ^41^, ^42^, ^43^ STAT1 was upregulated by 9.43-fold in M1 macrophages and was associated with the upregulation of the corresponding target chemokines CCL19, CCL20, CCL4, CCL5, CCR7, CXCL10, CXCL11, CXCR3, and CXCL9. Moreover, interferon regulatory factor-1 (IRF-1) is an important nuclear TF with a variety of biological functions, such as promoting the systemic inflammatory response and regulating the development and polarization of immune cells.^44^ IRF1 was upregulated 18.75-fold in M1 macrophages and was associated with the upregulation of the corresponding target chemokines CCL19, CCL20, CCL5, CXCL10, and CXCL11. In addition, the miR-15 family consists of 6 highly conserved miRNAs (miR-15a/b, miR-16, miR-195, miR-497, and miR-322), and the genes that encode them are distributed on 3 different chromosomes.^45^ Among them, miR-15a can induce apoptosis and reduce vascular injury caused by ischemia by regulating the post-transcriptional level of Bcl-2.^46^, ^47^, ^48^ miR-21 is highly expressed in many tumours; is involved in cell proliferation, polarization, and apoptosis; and plays an important role in the development of tumours.^49^ By combining these results with previous results (Fig. 5), we obtained a model by which chemokines regulate macrophage polarization (Fig. 7).

**Figure 8:**
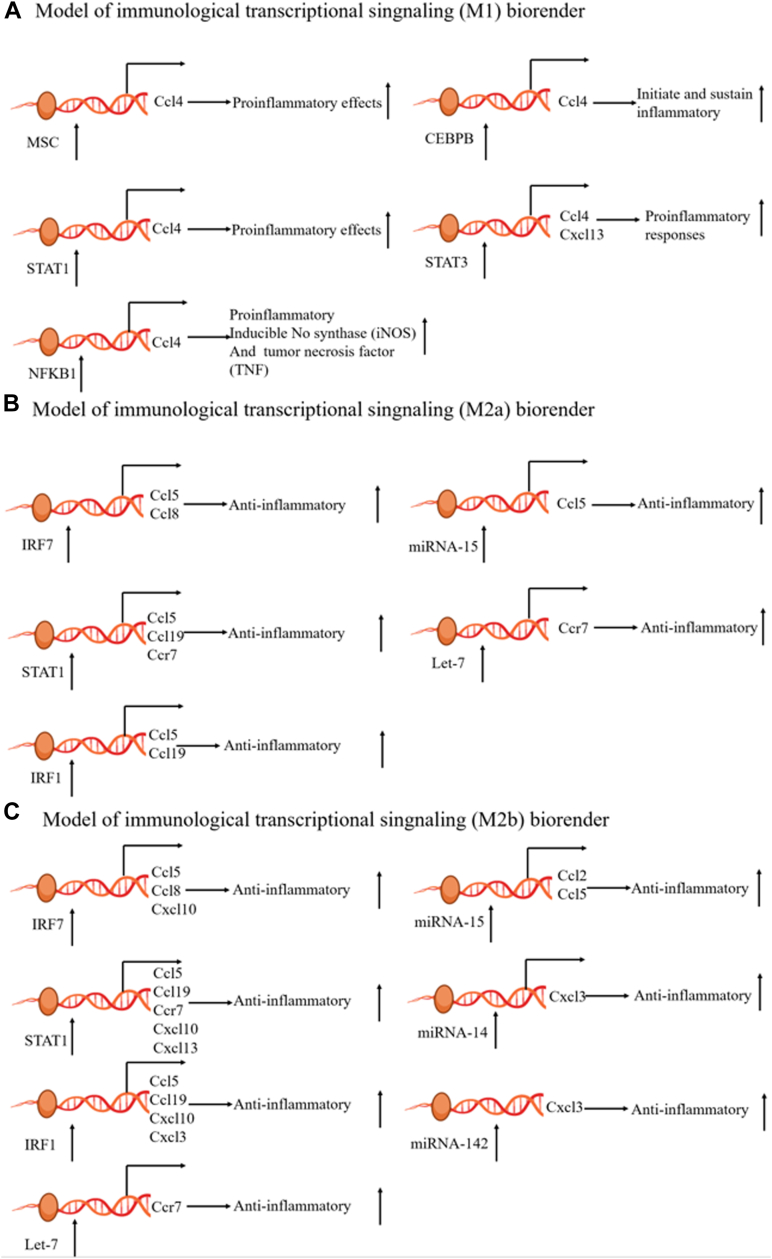
Model of chemokine regulatory atherosclerosis through macrophage polarization.

In Figure 8, we present a possible model of the regulation of AS by chemokines. Five of the SDE TFs (CEBPB, STAT1/3, NFKB1, and MSC) promote progression of AS by regulating chemokines (CCL4 and CXCL13) that promote M1-type macrophage polarization. Three SDE TFs (IRF1/7 and STAT1) promote AS progression by regulating chemokines (CCL5, CCL8, and CCL19), and 3 SDE TFs (IRF1/7 and STAT1) inhibit progression of AS by promoting M2a macrophage polarization through the regulation of chemokines (CCL5, CCL8, CCL19, CXCL3, CXCL10, and CCR7). Two SDE miRNAs (Mir-15 and Let-7) promote M2a-type macrophage polarization through the regulation of chemokines (CCl5 and CCR7), thereby inhibiting the progression of AS. Three SDE miRNAs (Mir-14, Mir-142, and Let-7) promote M2b-type macrophage polarization through the regulation of chemokines (CXCL3, CCR7), thereby inhibiting the progression of AS.

## Discussion

Chemokines are now known to play an important role in the entire process of formation of AS. However, the mechanisms by which chemokines induce macrophage polarization and regulate formation of AS are unclear. In this study, we investigated their transcriptional regulatory pathways and their functions through intensive bioinformatic analyses and literature searches. We have 5 major findings: (1) Chemokines are differentially expressed in mouse macrophages derived from different tissues. (2) Chemokines promote the polarization of primary macrophages into M1-, M2a-, and M2b-type macrophages. (3) TFs and miRNAs can modulate macrophage polarization by regulating chemokines. (4) Chemokines are differentially expressed in the progression and regression of AS. (5) Two sets of hypothetical molecular signalling models were developed. The first model describes the mechanisms of chemokine-mediated transcriptional regulation of macrophage polarization. The second model summarizes the potential molecular mechanisms underlying chemokine regulation of AS. Our study aims to elucidate the mechanisms by which chemokines regulate formation of AS and thus provide new targets for the treatment of the condition.

Previous studies have shown that chemokines can modulate AS by altering the number of circulating monocytes, inducing monocyte adhesion, and avoiding inhibition of macrophage apoptosis in atherosclerotic lesions.^26^^,^^50^, ^51^, ^52^, ^53^, ^54^ Similarly, our studies suggest that chemokines may also modulate the progression of AS by regulating macrophage polarization. Notably, this result is consistent with our chemokine GO and KEGG analyses (Fig. 2C). Considering the high risk of using chemokines alone to treat inflammatory diseases such as AS, which may lead to a decrease in host immunity, it is important to determine the exact role of chemokines in the development of AS. Specific chemokines involved in AS and their corresponding molecular signalling pathways may be targetable by new treatments.

There were differences in the expression of chemokines among 7 types of tissue-specific macrophages in mice. Of the 45 chemokine genes, only 7 chemokine genes were expressed in mouse tissues: CCL2, CCL4, CCL5, CCL6, CCL9, CCR2, and CX3CR1 (Fig. 3). This suggests that these 7 genes may function as housekeeping genes for mouse tissue macrophages. We found that some chemokine genes were only expressed at low levels in 1 tissue but were expressed at high levels in other tissues. For example, CCL7 was only expressed at low levels in splenic macrophages and was highly expressed in other tissues. In addition, we also found that some chemokines were only highly expressed in certain tissue macrophages, such as the chemokine CCL20, which was only highly expressed in colonic macrophages. These results suggest that the expression level of chemokines is related to the type of macrophages in tissue, and modulating the expression of chemokines may change the levels of macrophages. Generally, activated macrophages can be divided into M1, M2a, and M2b types. Through analysis of chemokine expression levels in macrophage polarization, we found that there was differential expression of chemokines in macrophage subtypes. For example, CCL2 and CCL11 were only highly expressed during M0 to M2b macrophage polarization, whereas CCL16 was only highly expressed during M0 to M1 macrophage polarization (Fig. 5). These results suggest that chemokines can regulate the polarization of macrophages. It is worth mentioning that macrophages are closely related to various inflammatory diseases. Therefore, understanding the upstream regulation and targeting factors of chemokines, and being able to modulate the polarization of macrophages in vivo by regulating chemokines, is important for the treatment of inflammatory diseases.

Chemokines play an important role in AS plaque diseases (Table 2), but how the progression of AS plaques is regulated is not completely clear. Previous studies have shown that chemokines regulate inflammation via miRNAs and TFs^55^, ^56^, ^57^, ^58^; therefore, we have reason to believe that chemokines are also regulated by miRNAs and TFs in AS lesions. To develop a chemokine-regulated AS model, we first determined the expression levels of 33 AS-related chemokines in AS plaques by scRNA-seq. The results showed that the chemokines were expressed at the start of both the progression and regression of AS plaques, but the expression levels were different. Some chemokines seemed to promote the progression of AS plaques, whereas others seemed to inhibit the progression of AS plaques. For example, CCL12 was more likely to promote the progression of AS plaques, while CXCR4 was more likely to inhibit the development of AS plaques (Fig. 4). Next, using IPA, we found that 10 SDE TFs (STAT/3, IRF1/7, RELA, NFKB1, NFKBIA, PML, CEBPB, RELB, NFKB2, and MSC) may be involved in the polarization of M1 macrophages (Fig. 6A). Our data suggest that 10 SDE TFs (STAT/3, IRF1/7, RELA, NFKB1, NFKBIA, PML, CEBPB, RELB, NFKB2, MSC) may be involved in the process of M1-type macrophage polarization (Fig. 6A). Among them, NFKB, RELA, RELB, STAT1, and STAT3 have been reported to promote M1 macrophage polarization through the regulation of chemokines and thus exert a proinflammatory effect.^26^^,^^59^, ^60^, ^61^, ^62^ Four SDE TFs (STAT1, IRF1/7, Bcl-3) may be involved in the polarization of M2a and M2b macrophages (Fig. 6A). To further clarify the mechanism by which TFs regulate macrophage polarization through the modulation of chemokines, we combined the results from this study with our previous results (Fig. 5), and further obtained a model in which TFs regulate macrophage polarization through the modulation of chemokines (Fig. 7). Moreover, we also obtained a model in which miRNAs regulate macrophage polarization through the modulation of chemokines using the same methods (Fig. 7). Interestingly, based on our results, we have several new findings: The TF STAT1 can promote M1 and M2 polarization of M0 macrophages by regulating chemokines, whereas the TFs STAT1, IRF7 and IRF1 can regulate M2a and M2b polarization of M0 macrophages by regulating chemokines. However, the chemokines regulated by TFs in these processes are different (Fig. 7). For example, some TFs promote M1 polarization of M0 macrophages through CCL4, whereas CCL19, CCL5, and CCR7 promote M2 polarization of M0 macrophages. TFs can both promote and inhibit AS, whereas miRNAs can only inhibit AS. In our model of chemokine regulating AS (Fig. 8), we found that CCL4 and CXCL13 can promote the polarization of M0 to M1 macrophages; however, some studies^63^^,^^64^ have shown that CCL4 can not only promote AS but also delay AS, which suggests that CCL4 may play different roles because of different regulatory factors involved in the regulation of CCL4; CCL8, CCL19, CCL5, and CCR7 can alleviate AS by promoting the polarization of M0 to M2a and M2b macrophages, but some studies^64^^,^^65^ have shown that CCL5 and CCL8 can promote AS. In addition, some studies^66^^,^^67^ have shown that chemokine CXCL10 and CXCL3 play a role in the progression of atherosclerosis, but our study found that CXCL10 and CXCL3 play a role in alleviating AS only by promoting M0 to M2b macrophages and suggest that CXCL3 and CXCL10 may be important chemokines in promoting the polarization of M2B macrophages. This suggests that the role of CCL5, CCL8, CXCL3, and CXCL10 in AS needs further research.

**Table 2:** Chemokines that have been shown to be associated with the progression and regression of atherosclerosis

Chemokine	Role in atherosclerosis	PMID
CXCL13	Promoting	31837653
CXCL10	Inhibition	31115530
CCR7	Inhibition	16537455/ 22163030
CCL19	Inhibition	16537455
CCL4	Inhibition	32911750
	Promoting	21133894
CCL5	Promoting	21133894
CCL8	Promoting	33741452
CXCL3	Promoting	37121164

In this study, bioinformatics methods were used to analyze the expression levels of chemokines comprehensively in 7 mouse tissues and macrophage polarization for the first time and to further analyze the upstream regulatory factors of chemokines regulating macrophage polarization. In addition, we reported the expression levels of chemokines in the progression and regression of AS and analyzed the possible regulatory models and mechanisms of chemokines and macrophage polarization in AS.

### Limitations

There are some shortcomings in this study. For example, although we eventually developed a model in which chemokines modulate AS by regulating macrophage polarization, it seems more likely that AS progression is delayed by modulating CCl8 activation based on the results in Figure 4. In addition, although the results show that CCL4 is more inclined in atherosclerosis, CCL4 does not appear to be significantly different in progression and regression of AS plaque. These results were only obtained using bioinformatics analysis and require experiments for further validation. Chemokine pathways and their roles in macrophage polarization have not yet been tested in vivo or in vitro. Prospective studies are crucial to verify whether these bioinformatically derived mechanisms hold true in both physiological environments and human subjects. In addition, the complex interactions between chemokines and other signalling molecules in the human immune system may differ from those in the models analyzed, necessitating more comprehensive animal and human studies to validate the potential therapeutic implications. To solve this problem, future research should focus on validating the identified chemokine pathways in human tissue or in clinical settings. One approach could be to analyze chemokine expression in human atherosclerotic plaques for comparison with the murine models used in this study. Also, clinical trials involving targeted modulation of chemokines such as CCL4; CCL5; or CXCL3 through gene therapy, antibody treatment, or small molecule inhibitors could provide critical insights into their roles in human AS. Prospective trials should aim to determine whether these chemokine targets function similarly in humans, thereby bridging the gap between animal models and clinical applications.

## Conclusions

In summary, our bioinformatics analysis of chemokine regulation in AS revealed that treatment of AS can be achieved by modulating chemokines to regulate macrophage polarization. The chemokines CCL4 and CXCL3 may play key roles in the progression of AS. Chemokines CCL5, CCL8, CCL19, CXCL3, CXCL10, CXCL13, and CCR7 may play a key role in regression of AS. This study discussed not only the relationships among chemokines, macrophage polarization, and AS but also the multiple signalling pathways by which chemokines regulate AS by modifying macrophage polarization, providing new options for the treatment of AS.
